# An anthropomorphic phantom representing a prematurely born neonate for digital x-ray imaging using 3D printing: Proof of concept and comparison of image quality from different systems

**DOI:** 10.1038/s41598-019-50925-3

**Published:** 2019-10-07

**Authors:** Nikolaus Irnstorfer, Ewald Unger, Azadeh Hojreh, Peter Homolka

**Affiliations:** 1 Center for Medical Physics and Biomedical Engineering, Medical University of Vienna, Vienna, Austria; 2 Department of Biomedical Imaging and Image-guided Therapy, Medical University of Vienna, Vienna, Austria

**Keywords:** Biophysics, Biological physics, Applied physics, Radiography

## Abstract

An anthropomorphic phantom for image optimization in neonatal radiography was developed, and its usability in optimizing image acquisition and processing demonstrated. The phantom was designed to mimic a patient image of a prematurely born neonate. A clinical x-ray (neonate <1 kg) taken with an effective dose of 11 µSv on a needle-crystal storage phosphor system was retrospectively selected from anonymized images as an appropriate template representing a standard case in neonatology imaging. The low dose level used in clinical imaging results in high image noise content. Therefore, the image had to be processed using structure preserving noise reduction. Pixel values were related to printing material thickness to result in a similar attenuation pattern as the original patient including support mattress. A 3D model generating a similar x-ray attenuation pattern on an image detector as a patient was derived accounting for beam hardening and perspective, and printed using different printing technologies. Best printing quality was achieved using a laser stereolithography printer. Phantom images from different digital radiography systems used in neonatal imaging were compared. Effects of technology, image processing, and radiation dose on diagnostic image quality can be assessed for otherwise identical anthropomorphic neonatal images not possible with patient images, facilitating optimization and standardization of imaging parameters and image appearance.

## Introduction

Neonatal imaging is in many respects different to adult and general pediatric radiography. Imaging of small prematurely born neonates with weights that may be as low as 500 or 700 grams is especially challenging since structures to be visualized are small, and contrasts are low. This puts high demands on both, detectors used, and digital image processing. Regarding the former, the choice of hardware includes technologies with very different characteristics, as simple powder type storage phosphor systems, needle type storage phosphor plates, or portable flat panel detectors. Image processing is often not optimized for this very special patient class, und sub-optimal image processing may - and will - result in inferior image quality, or unnecessary high patient doses. Additionally, commercial image processing algorithms implemented into the radiography systems are usually black boxes and often used with vendor settings rather than being optimized for neonatal imaging^1^. These image processing algorithms significantly impact on perceived, i.e. diagnostic, image quality. This results in inferior diagnostic quality in some systems compared to others, if standard settings are employed^1^. However, optimization and even matching of different algorithms and imaging hardware to result in comparable image appearance is a difficult task^2^. Differences in perceived image quality, i.e., image quality ratings by the readers between different commercial image processing systems are typically more pronounced for lower doses than for higher dose levels^1^. This indicates, that images taken with lower doses require more sophisticated image processing to be accepted as diagnostically appropriate. Neonatal imaging at dose levels as presented in this paper represent some of the lowest doses in imaging since patients are very sensitive to  radiation, and usually receive repeated x-ray examinations, and thus optimization of image processing is of utmost importance.

Different approaches have been employed in optimization and matching of exposure settings and image processing. The first possibility is to use semi-anthropomorphic stylized phantoms like the Gammex 610 neonatal chest phantom (Gammex, Sun Nuclear, Melbourne, FL, USA)^1,2^. This type of phantom allows measurement of technical image parameters like signal to noise rations (SNR) or contrast to noise (CNR) in grossly simplified stylized anatomic structures. However, these structures cannot be regarded patient equivalent in either anatomic noise, fidelity, or spacial frequency content. It is well documented that physical parameters like effective detective quantum efficiency (eDQE) or CNR/SNR are not an adequate predictor of diagnostic image quality^3^. Another possibility employed by a different group is using animal models. Conradie and Herbst used five rabbits as a model animal representing prematurely born neonates to optimize dose level in neonatal chest imaging. They concluded that a dose reduction from their previously used protocol was feasible^4^. However, interobserver variability in judgement of image quality features was high. The last approach for comparing image quality obtained with different protocols used clinical patient images where neonates where assigned to either protocol randomly, and alternating protocols for an individual patient for repetitive follow-up images using a visual grading scheme for seven image features^5^. The last two approaches actually target clinical image quality; however, direct comparison of otherwise exactly identical images is not feasible.

These issues could be overcome, if phantoms producing absolutely identical patient equivalent radiation patterns on any image detector were available. Images of these phantoms could be used for direct side-by-side comparison between different detector systems, different image processing algorithms on the same or on different systems, and different acquisition settings. However, this approach would necessitate the availability of a truly anthropomorphic phantom producing patient-like x-ray images on an x-ray detector. Optimally, these phantoms would mimic individual selected patients allowing a set of phantoms representing simple or difficult clinical conditions and a variety of neonate weights. The aim of this work was to investigate whether such a phantom can be produced using a standard commercially available 3D printer and regular clinical low dose patient images as printing templates.

The need of optimization for this special patient population has already been emphasized above. If image acquisition is optimized according to local clinical needs, dose levels can usually be corrected downwards. It is a commonly seen approach that non-optimal image processing is compensated for by using higher dose levels. This reflects that the large potential provided by advanced image processing that is typically vendor specific and parametrized by variables difficult to understand by the end user^6^ is often left fallow. Additionally image processing is intimately associated with exposure conditions^6^. This requires image processing be adapted if exposure conditions, like beam hardness, are changed.

Having otherwise absolutely identical raw images would thus greatly simplify optimization of processing and cross-platform harmonization of image appearance. In case non-local contrast amplification like multifrequency processing or tissue or anatomy identification or segmentation-based processing is used, it is imperative that the images resemble a clinical image as closely as possible in both, real and frequency space.

### 3D Printing

Three-dimensional printing is applied in many fields in medicine, ranging from production of customized surgical tools and prostheses, implants or dental restorations, operative rehearsal to visualization of complex surgical procedures^7,8^. Another opportunity, especially when controlling radio opacity by either material selection^9–11^ or by customizing printing materials to exhibit x-ray attenuation properties desired^6,12–14^ is the production of phantoms for dosimetry^15^, quality assurance^16,17^ and similar applications.

In the fabrication of phantoms, the two most commonly applied printing technologies are either fused deposition modeling (FDM) or stereolithography (SLA) printing. In an FDM system a thermoplastic material usually supplied as continuous filament is melted in a printer extruder and deposited on a horizontal plane where it cools and solidifies. In SLA systems a light source, usually a UV laser or projector, initiates photopolymerization in a liquid resin resulting in solidification. Both technologies may be used, however in standard commercial systems typically SLA produces higher detail accuracy and smoother surfaces at higher cost compared to FDM.

### Phantom design

Different local patient attenuation resulting in the x-ray contrast in the radiation pattern on the detector and consecutively in the x-ray image as different grey values were transformed into corresponding material thicknesses in the phantom. Local higher attenuation in the patient results in longer attenuation length in the phantom.

**Figure 1:**
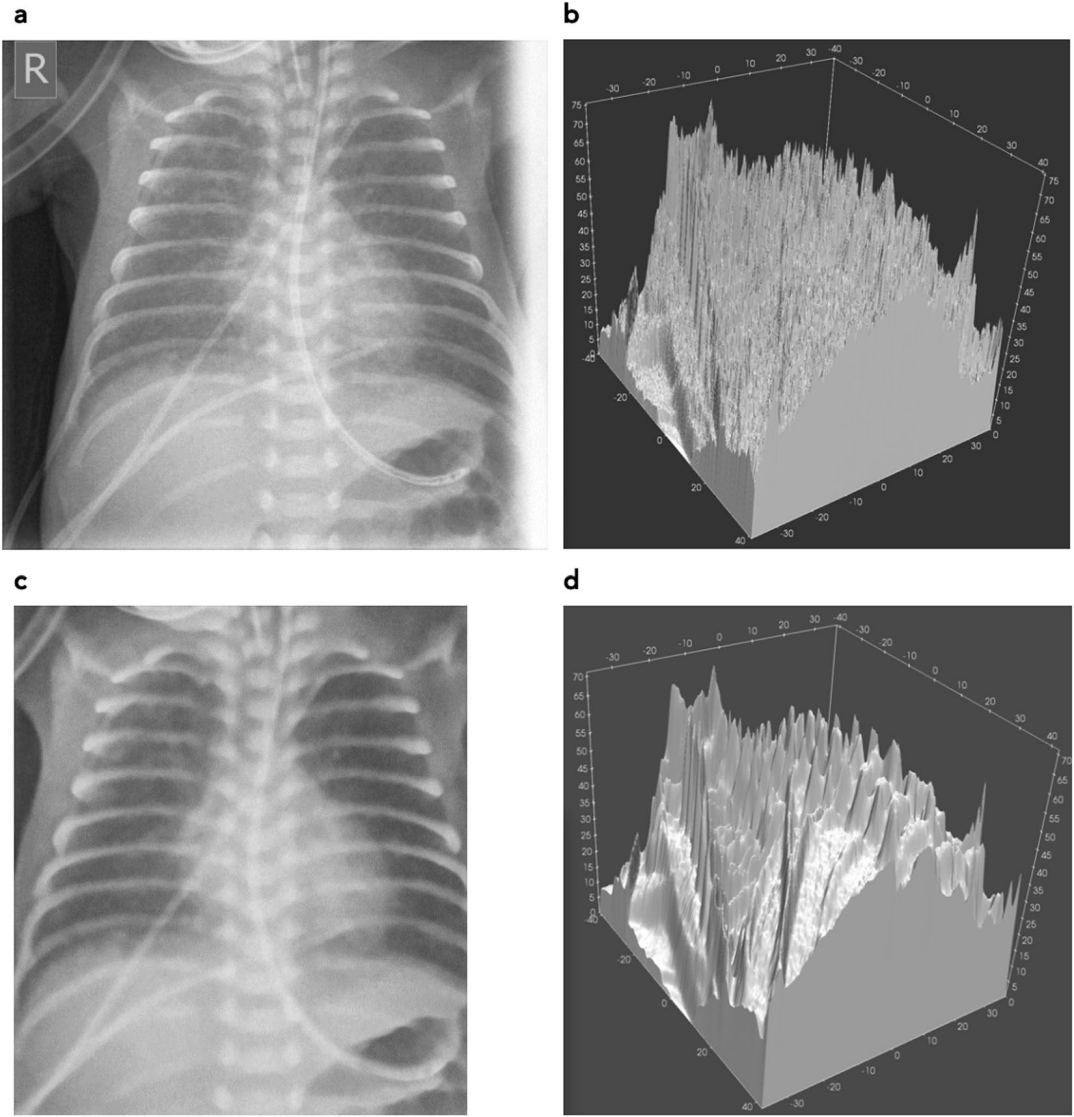
Patient image used as printing template, and 3D visualization of the respective phantom STL models before and after de-noising. (**a**) Diagnostic patient image without noise reduction, and (**c**) after cropping and noise reduction. Corresponding phantom STL models are shown in (**b**,**d**).

One of the biggest challenges in printing such phantoms from regular patient images is image noise. This noise is mostly due to quantum noise since the patient images are taken with as low dose as necessary for appropriate clinical image quality. Before printing, this image noise needs to be reduced greatly using edge preserving de-noising algorithms, because the phantom shall mimic the patient attenuation rather than the noisy image. If a phantom with noise left in the printing template would be imaged with the same protocol as a patient, the noise left in the phantom, and the quantum noise from imaging would add up. This would result in different noise level and noise texture compared to patient images, and would greatly reduce the usability of the phantom. On the other hand, noise reduction without sacrificing some detail and fidelity is not possible. Therefore, a balance between residual noise content, and preservation of detail and structure must be found. Figure 1 illustrates the noise reduction by comparing images and resulting 3D phantom for the original noisy image, and after noise reduction, respectively.

In order to allow a correct image noise measurement in the phantom a homogeneous region with a diameter of 7 mm was added in the liver region. The original patient image also had to be cropped to remove image parts affected by penumbra from collimation. Figure 1 shows the original patient image (a) and the image after cropping and noise reduction as used to print the phantom (c). Figure 1(b,d) visualize the resulting phantom if the corresponding images were used as printing templates translating pixel values to polymer thickness. Using the original image with image noise as seen in the patient image thus would result in an unprintable STL file (b). The corresponding printing file after noise reduction is shown in (d). From Fig. 1(b) it is obvious, that a high level of detail preserving noise reduction is necessary to produce a usable printing template. Dimensions of the phantom are 83.6 × 74.0 mm and 71.1 mm maximum height.

## Results

**Figure 2:**
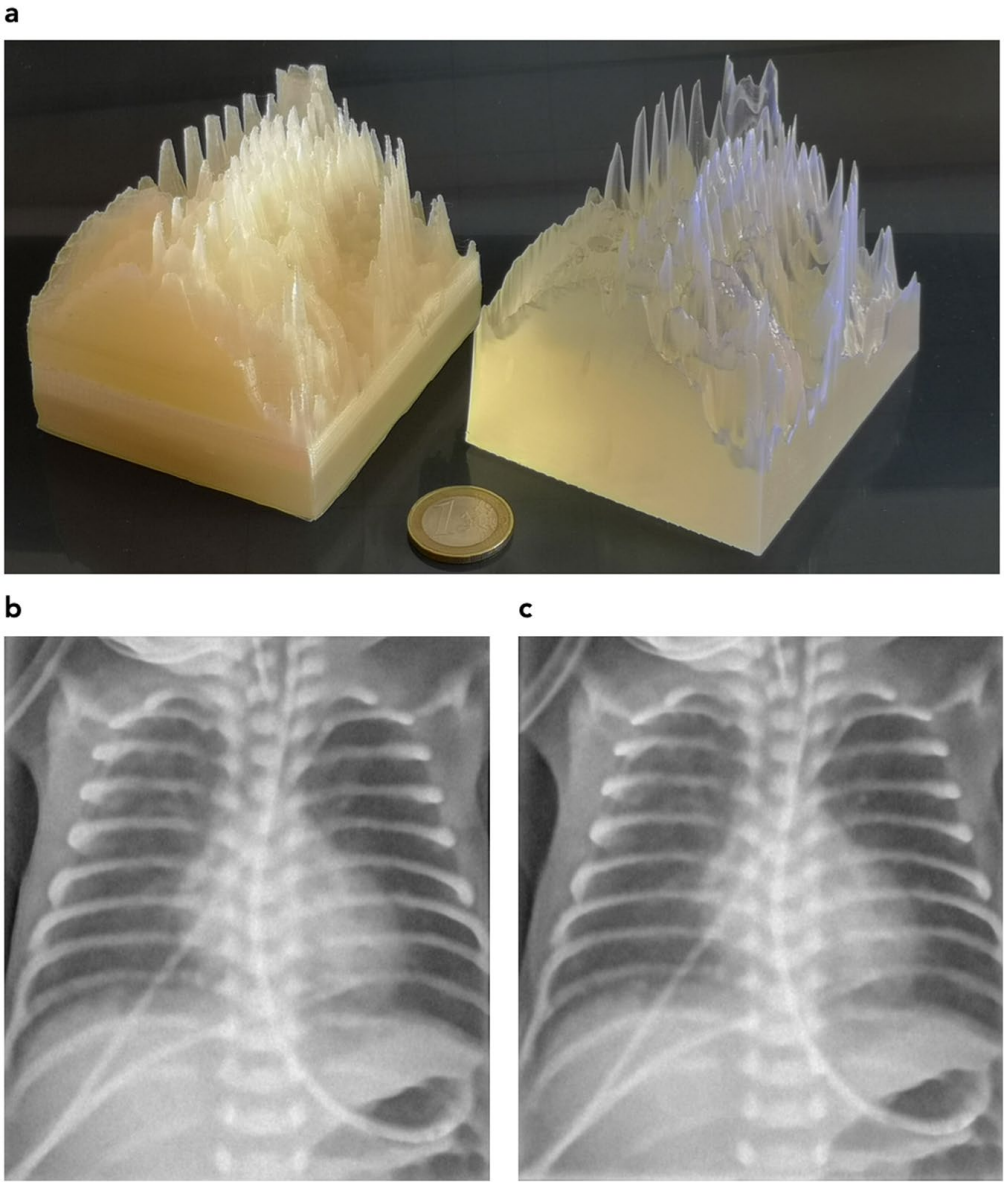
Phantoms printed with FDM (left) and SLA (right image) technology, respectively (**a**), and corresponding x-ray images (**b**: FDM, and **c**: SLA). Better reproduction of sharp structures and fine details with SLA is evident.

Comparison of printing technologies reveals the superiority of stereolithography as opposed to fused deposition modeling in reproducing small details. FDM also showed tendencies of stringing and warping. However, these could be minimized by choice of printing polymer and temperature but still should be considered a factor limiting reproduction of very fine details and/or solid printouts. Figure 2 compares the results from both printing technologies. In (a) the printouts are directly compared where the better reproduction of sharp edges and small details with the SLA printer is evident. (b,c) depict the corresponding x-ray images (Agfa storage phosphor system, DX-G/S170-100/CR HD5.0). Better reproduction of details can be seen in the SLA printout e.g. in the catheter walls in the upper left corner of the image, and in the reproduction of the spinous processes. Therefore, the SLA print was chosen for further evaluation and subsequent use as phantom to be used for optimization exercises in clinical systems.

Effects from noise reduction are evident in both printouts. They can already be seen in Fig. 1 comparing (a,c).

### Comparison of template image, de-noised patient image and phantom images

De-noising of the original patient image (Fig. 1(a)) reduces fine structures corresponding to high spacial frequencies despite application of structure conserving de-noising algorithms. This limitation can only be overcome by using high dose x-ray images instead of low dose patient images requiring using animal models instead of patients. The effect can best be visualized by comparing Figs 1(a) and 2(c).

**Figure 3:**
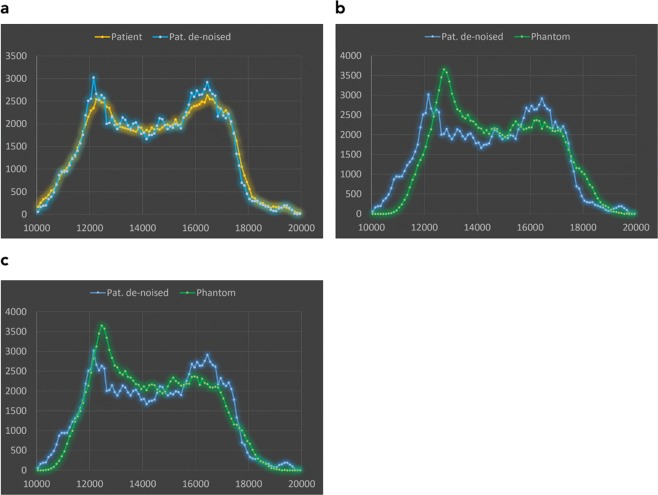
Comparison of image histograms: (**a**) original patient image compared to de-noised image used as printing template. (**b**) de-noised patient image used as printing template compared to phantom image taken with same system at identical exposure and image processing parameters, and **(c)** shifted by 300 PV (pixelvalues).

**Figure 4:**
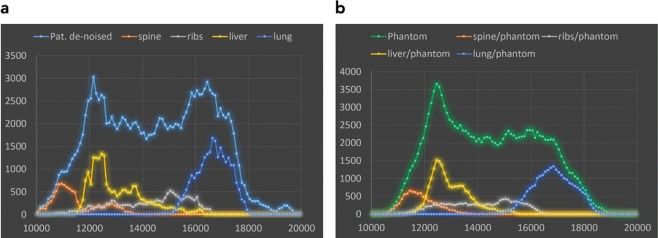
Pixel value histograms of patient (**a**) or phantom (**b**) respectively, and histograms of pixels belonging to spine, ribs, liver and lung tissue.

**Figure 5:**
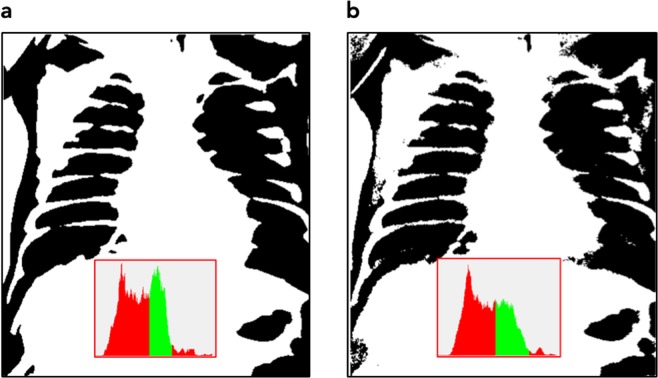
Thresholded binary images indicating pixel intensities corresponding to lung tissue (black). Threshold settings correspond to green part in the histograms. (**a**) de-noised patient image, (**b**) phantom image.

Pixel value histograms of the original patient image, the de-noised patient image and the x-ray image of the phantom taken with identical settings on the system used to acquire the original patient image (Agfa needle type storage phosphor system) are compared in Fig. 3. As seen in Fig. 3(b) the histogram of the de-noise patient image is slightly shifted towards higher pixel values by the clinically used image processing due to automatic signal normalization setting the median at identical values. This is a result from a different form of the histogram in the lung region. In Fig. 3(c), this shift has been corrected (by subtracting a value of 300) to better demonstrate concordance and differences in the histograms. These differences can best be demonstrated and understood by evaluating histograms analyzing pixel values in different tissues or organs. In Fig. 4(a,b) pixel value histograms from pixels corresponding to the spine, the ribs, liver and lung tissue are shown together with the image histograms from Fig. 3(c). While the other tissues/organs present with similar distributions, pixels corresponding to lung tissue present with a wider distribution and thus lower maximum, since the area under curve corresponding to the number of pixels segmented as lung tissue, is equal in both images. This effect can also be anticipated when comparing the phantom image with the de-noised patient image. It is best demonstrated in Fig. 5. Here, thresholded binary representations of the de-noised patient image (a) and the corresponding phantom image are shown. Threshold values were set to segment lung tissue; however, all other pixels with pixel values in the corresponding range are also included and represented in black. The part of the corresponding histograms between the thresholds are shown in green. It can be seen, that in the phantom image (b) pixel values corresponding to lung tissue use a slightly wider portion of total grey values.

### Comparison of different x-ray systems

In addition to the Agfa needle type storage phosphor system the phantom has been imaged on 3 other systems used in pediatric radiography applying portable indirect flat panel detectors at a slightly higher dose level corresponding to typical exposure settings in these systems.

**Figure 6:**
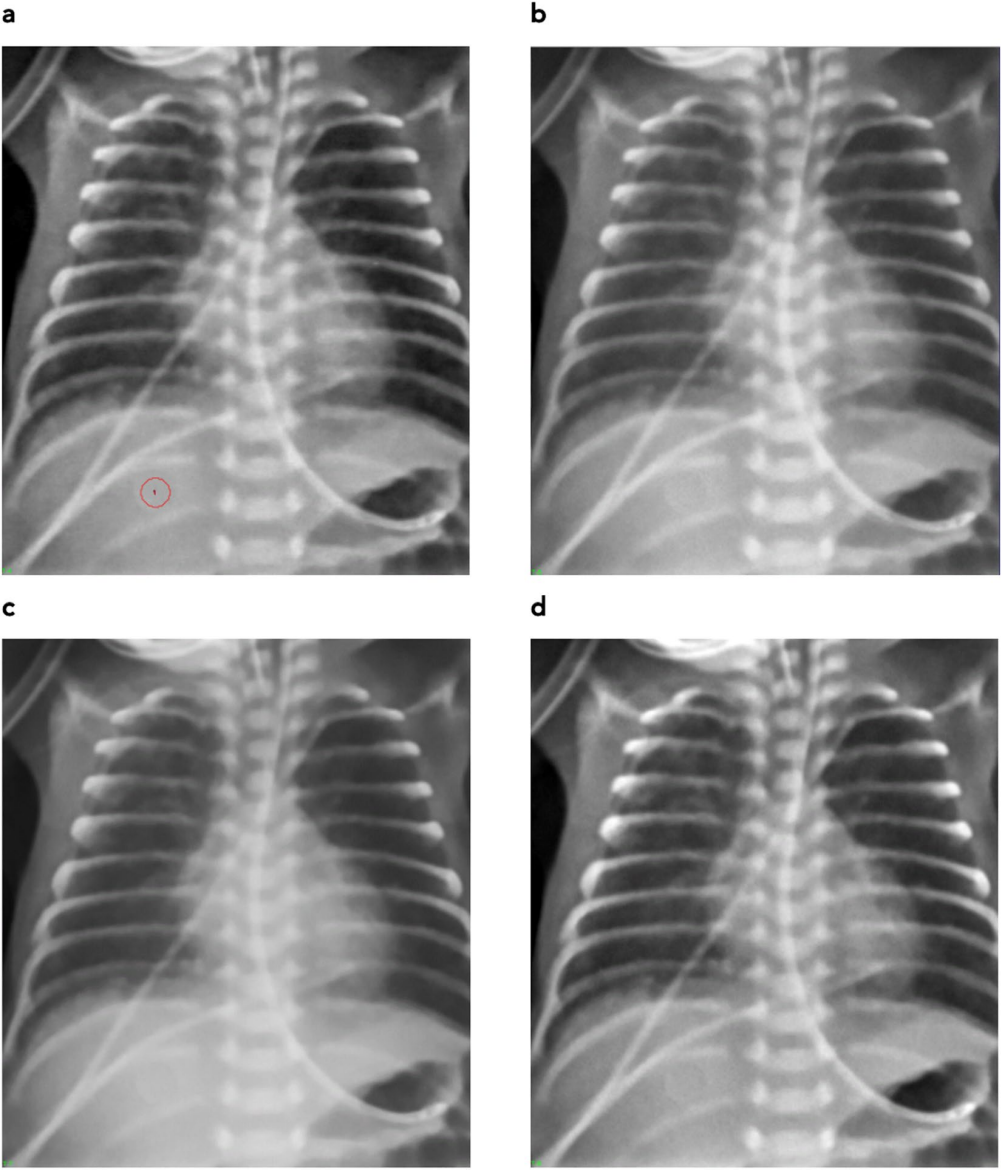
Phantom images from different systems normalized to identical level and latitude. (**a**) Agfa needle type storage phosphor (reference system), (**b**) system 2, (**c**) system 3, and (**d**) system 4. In (**a**) ROI in the homogeneous disk in the liver region used for noise measurements is marked (red circle).

**Figure 7:**
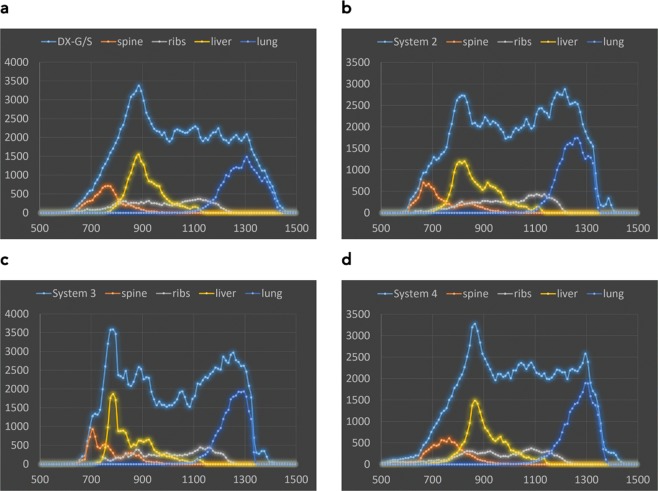
Histograms corresponding to images in Fig. 6. (**a**) Reference system Agfa DX-G/S needle type storage phosphor, (**b**) system 2, (**c**) system 3, and (**d**) system 4.

The resulting images and histograms are provided in Figs 6 and 7. Latitude and median were both normalized to 1024 to ensure equal window settings, and allow direct comparison with identical windowing.

Phantom images taken with different clinical systems at identical beam quality and dose level demonstrate company specific differences in image processing algorithms seen accordingly in clinical images. Understanding these differences greatly helps radiologists to express their requirements in optimizing image processing, and exposure settings, and facilitates cross-platform image standardization. Comparing images from Fig. 6 reveals differences in contrast (e.g., lung contrast) and noise content despite equal dose and presentation setting, i.e. level and latitude. This is reflected in the histograms (Fig. 7) and signal to noise ratios. Table 1 shows image noise measured as standard deviation (STD) and signal to noise ratio (SNR, defined as mean pixel value divided by the STD) measured in the homogeneous disk placed in the liver region of the phantom. The corresponding region of interest used on all phantom images is marked exemplary in Fig. 6(a).

**Table 1:** Noise and signal to noise ratio (SNR) measured in the homogeneous disk of the phantom in the liver region.

	Noise	SNR
DX-G/S	11.1	77.1
System 2	15.4	51.7
System 3	4.3	183
System 4	10.9	78.4

Using these phantom images, differences in image processing and diagnostic image quality can clearly be seen between different systems. All systems were applying image processing settings advised by the corresponding vendor for use in neonatal radiography. Systems 2 and 3 use image processing algorithms that result in better harmonization of grey values between soft tissue, bone and liver corresponding to lower Shannon entropy^18^ than seen in the other two images. In system 2 this is also due to an advanced local contrast enhancement. The latter is also seen in the reference systems (DX-G/S) and system 4 in enhanced local contrast of the vertebrae against the liver. The image from system 3 appears softer with regard to contrasts and noise, compared to the others. In the histograms this corresponds to a smaller portion of the latitude (lesser gray values) allocated for bone (spine) and soft tissues (liver), and the widest gap between high attenuation (spine/liver regions) and the lung. In addition, image sharpening is also markedly less, seen in both the image and the noise. Signal to noise levels (measured in the liver region) are 2.3 to 3.6 times larger than in the other systems. Comparing image impression and histograms between the reference system and system 4 exhibits similar histograms and thus similar image characteristics, with a difference in the depiction of lung tissue. Corresponding to the slightly narrower histogram of lung tissue in system 4, which exhibits a markedly steeper flank on the high pixel value end, the lung appears with a little more condensed grey values and thus slightly poorer depiction of internal structures. Noise levels are equivalent.

## Discussion

Overall image impression with regard to contrasts were similar between the phantom image and the image template corresponding to the de-noised patient image. Therefore, the phantom can be regarded “anthropomorphic” with regard to overall tissue structures (soft tissue/bone) and contrasts. However, a minor systematic difference was found in the lung contrast, where the lung region has a wider latitude in gray values in the phantom image as compared to the printing template. This is be seen in comparison between Figs 1(c) and 2(c), and the corresponding histograms in Figs 3(c) and 4. This is most likely due to the clinical image processing (IP) applied. This may correspond either to a non-linear gamma correction assigning different gamma values in brighter and darker regions, and/or frequency-based IP. The former is commonly used to either compress or increase contrasts specifically in the dark or light parts of an image. Another image processing resulting in these effects can be non-local frequency-based IP as implemented by the Agfa system used and known as MUSICA (multi scale image contrast amplification) with Fractional Multiscale Processing^1,19^. This finding indicates, that the (black box) clinical image processing has increased contrasts in the original patient image used as printing template, and again in the processing of the phantom image. This most likely results in an over-processing in the lung. Another issue that might also contribute results from the different frequency spectra in the lungs between original image and phantom image. Since the original image needs to be de-noised, high frequency bands are strongly damped losing fine structure in the lungs, which in return will result in differences in a frequency-based IP algorithm for lung tissue.

### Limitations of this study

One of the main limitations of this study results from the necessity to de-noise the clinical image used as printing template. Since patient images taken at extremely low doses exhibit high quantum noise, however, noise removal has to be applied. Different algorithms also used in clinical image processing have been developed to best preserve structure. Denoising, even if structure preserving, always degrades detail. In this work black-box algorithms implemented into a professional software package optimized for digital photography have been used. Lung structure and bone contours are affected by these algorithms. Also, visual inspection of critical structures as opposed to quantitative measures were applied to define the level of noise reduction to be used in the printing template. However, deciding on the level of de-noising remains subjective because limited by the accepted remaining noise, and acceptable loss of detail. To study image processing using images of this phantom in clinical settings, and optimize settings of spatial frequency-based image processing, a realistic lung structure would be extremely useful. Due to the layout of this study, i.e. using a (ultra)low dose patient image as printing template, this cannot be achieved. Therefore, future development of these phantoms should consider integration of some kind of realistic fine-structure in the lungs mimicking also spectral properties of the patient lung.

Directly related with the use of real patient images as printing template is the second limitation. Patient images are always processed with (optimally custom tailored) algorithms. Also, in this study the patient image has been processed with clinical image processing optimized for neonates. In a clinical imaging department, raw images (or DICOM for processing images) are not available to the end user. In further *prospective* studies raw images (DICOM for processing) could be secured and used, and image processing optimized for 3D printing developed and applied, rather than using post-processing on images optimized for diagnostic image reading. However, the effect of the image processing was accounted for by the calibration procedure as appropriately as possible. Nevertheless, image processing of the Agfa DX-S used in this study includes latitude adaption besides automatic signal normalization. This image processing steps will normalize images within a wide range of latitudes in the radiation pattern to the same image latitude. Therefore, the calibration procedure requires a dynamic range and thus appropriate minimum and maximum step heights in the step wedge used similar to the patient. This fact has been considered when designing the corresponding step wedge and calibration procedure. However, access to raw or DICOM for processing images would alleviate this issue.

Lastly, due to the 2.5-dimensional nature of the phantom as opposed to a truly 3-dimensional patient or object, x-ray images of the phantom need to be taken well centered and with a source detector distance close to the one used in the calculation of perspective correction (i.e., 100 cm plus/minus 5 cm).

## Methods

### 3D Printers and printing materials

The printers used in this study were a FDM system (Ultimaker 2+, Ultimaker B.V., Geldermalsen, The Netherlands) and an SLA printer (Formlabs Form 2, Formlabs Inc., Somerville, MA, USA). Several printing materials for both technologies have been evaluated for the production of the phantom described in this work. The optimum material with regard to achievable print quality for the neonatal phantom in the FDM system used to print the phantom was polylactic acid (PLA F1906 transparent, Ultimaker B.V.). Other materials tested with favourable x-ray attenuation and energy dependence properties were polypropylene, polyamide, and acrylonitrile butadiene styrene (ABS). However, PLA exhibited least issues with stringing and warping in our printing platform compared to these. Other candidate materials having yielded good results include acrylonitrile styrene acrylate (ASA) and Polyethylene terephthalate (PET) co-polyester.

In the SLA system standard clear photocurable resin (Clear Resin V04, Formlabs Inc.) was used since chemical composition is best documented for this resin system. For the SLA printer the only user selectable parameter influencing the final outcome is layer height, which was set to 0.05 mm. Sine the SLA printer used in this work does not allow the use of custom resins, the choice of printing material is limited. In open resin printing systems, acrylic resins from different vendors exhibited similar results.

With the FDM printer a standard 0.4 mm Olsson block brass nozzle was employed. The first layer was extruded with 235 °C and then printing temperature was reduced to 215 °C. Infill was set to 100%, layer height to 0.1 mm and printing speed to 60 mm/s. Printing times were 87 hours and 20 hours, for FMD and SLA; printing material used were 257 grams (PLA, FDM print) and 249 ml resin (SLA), respectively.

### X-ray systems and printing template

X-ray imaging of neonates is performed on a needle crystal CR system (Agfa DX-G/S170-100 with CR HD5.0 detector 24 times 30 cm, Mortsel, Belgium). Protocols applied depend on weight of the patient. The patient image selected as printing template is shown in Fig. 1(a). It corresponds to a neonate with a mass of 880 grams weight who was intubated with a gastric tube. ECG electrodes can be seen on the image. Exposure parameters applied were 48 kVp, 0.9 mAs, no added filtration. Incident air kerma was retrospectively determined by measurement with a calibrated semiconductor dose multimeter (Unfors XI, Unfors RaySafe AB, Billdal, Sweden) as 17 µGy assuming 90 cm source skin distance corresponding to the standard procedure. This corresponds to an effective dose of approximately 11 µSv (PCXMC 2.0, STUK, Helsinki, Finland). The dose level can be regarded low and thus the image as a low-dose neonatal radiograph, compared to literature values of entrance surface air kerma values of 40 µGy after optimization in one study^5^, and median effective dose per chest radiograph of 13.3 µSv in another^20^. Monte Carlo Calculation of effective dose was carried out using the ICRP 103 definition^21^ of effective dose on the baby phantom scaled to 800 grams. Anatomical landmarks corresponding to the patient image were used to define collimation. According to university policies retrospective use of anonymized clinical images for research proposes necessitates clearance by the institutional data protection committee which was obtained prior to this study.

### Image de-noising

Since clinical images are acquired with minimum patient doses allowing sound diagnosis x-ray images are afflicted with high Poissonian image noise. Since achievable printing resolution is less than the original pixel dimension of 0.1 mm in x and y dimension, the image was resampled by averaging 2 pixels in both dimensions reducing noise by pixel averaging in a first step. In a next step, image noise had to be reduced further by applying edge preserving noise reduction algorithms. Adobe (Adobe Systems, San José, CA, USA) structure preserving noise reduction algorithms implemented in Photoshop CS6 were used since readily available and likely the most commonly-used noise reduction filter in digital image processing. Appropriate results were achieved applying the *Reduce Noise* filter twice with strength set to 10 and preserve details to 100%. Since the resulting image still contained a too high noise level to allow printing of a smooth phantom, noise was further reduced applying the *Remove Noise* (Photoshop CS6) filter repeatedly. After each application of the filter important image structures (catheters, lung, ribs, organ contours) were visually inspected to find the optimum balance between noise reduction and loss of image structure. This was found for 10 iterations of the *Remove Noise* filter.

### Calibration of pixel values to phantom height

**Figure 8:**
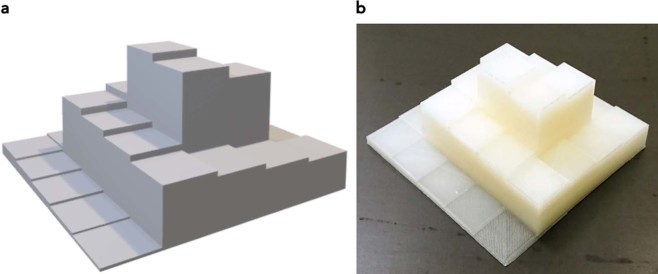
3D model and printout of step wedge used to relate pixel values to material thickness. (**a**) model, and (**b**) printout.

Lower pixel values corresponding to larger local x-ray attenuation correspond to a longer attenuation path in the polymer phantom. However, since the CR image used as printing template does not provide quantitative information as would a CT image, pixel values need to be correlated with attenuation. This correlation is not simple or straight forward since most CR systems use either a logarithmic or square root readout in combination with a sigmoidal dynamic range compression. Since these algorithms are usually not available to the end user and may also depend on the image content or histogram, a direct calculation of attenuation from pixel values is not possible from processed (DICOM for presentation) images. Calibration was performed by printing a step wedge pyramid phantom (Fig. 8) from the same materials than the phantom was to be produced later with step heights from zero to 50 mm. These pyramids were imaged with identical exposure factors and image processing as the original patient image used as template on the needle crystal CR system (Agfa DX-G). Pixel values of the steps were measured and correlated to the respective material height using 3^rd^ order polynomial regression analysis. The resulting regression function was used to relate pixel values in the image to corresponding material height in the phantom. Since the exact same materials were used in the calibration step wedge and the final anthropomorphic neonate phantom, the calibration procedure accounts for beam hardening effects due to the spectral nature of the x-ray beam and the gamma correction curve applied by the image processing.

### Perspective and projection geometry

X-ray imaging corresponds to a central projection with a typical focus detector distance between 0.6 to 2 meters. In neonatal imaging, typical focus detector distances are approximately one meter. This results in a pronounced perspective in the image due to the three-dimensional nature of the patient (and the phantom, respectively) due to larger magnification of structures further away from the image detector than those closer. Since in the phantom different attenuations are produced by differences in material thickness, i.e., differences in attenuation path lengths, the divergence of beam paths must be accounted for. This is done by assuming a focus detector distance of one meter and a perfectly centered projection.

In a first step the corresponding attenuation path lengths in the phantom are calculated for every pixel of the image serving as template assuming parallel projection geometry. Afterwards, a perspective correction was applied. With *z* = *0* corresponding to the detector plane, and *z* = *f* to the focal spot, the magnification *M*(*z*) depends on the *z* coordinate. To compensate the magnification a point $\overrightarrow{x}$ of the surface of the phantom with distance *z* to the imaging plane the coordinates of $\overrightarrow{x}$ need to be transformed to ${\mathop{x}\limits^{\rightharpoonup }}^{\text{'}}$ according to

$${\mathop{x}\limits^{\rightharpoonup }}^{\text{'}}=(\begin{array}{c}{x}^{\text{'}}\\ {y}^{\text{'}}\\ {z}^{\text{'}}\end{array})=(\begin{array}{c}M(z)\,\ast \,x\\ M(z)\,\ast \,y\\ z\,\ast \,cos(\alpha )\end{array})$$

with

$$M(z)=1-\frac{z}{f},\,\sin (\alpha )=\frac{r}{\sqrt{{r}^{2}+{f}^{2}}},\,r=\sqrt{{x}^{2}+{y}^{2}}$$

After perspective transformation of the phantom the surface was triangulated to produce a stereolithography (STL, also known as standard triangulation or standard tessellation language) file for printing. The triangulation was simplified in order to reduce the number of vertices by removing unnecessary triangles and representing pixels by single points rather than squares with four vertices each.

### Comparison of x-ray images

**Figure 9:**
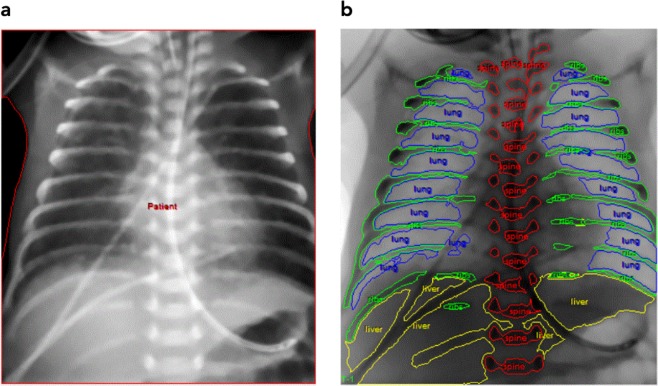
Segmentation and definition of ROIs used for calculation of histograms. (**a**) ROI used for total image histograms, and (**b**) segmented ROIs used for determining pixel value histograms for spine, ribs, lung and soft abdominal tissue in the liver region (the latter shown on inverted image for better presentation).

Pixel values and pixel value histograms have been compared between original and de-noised patient image, and phantom x-rays produced with different digital x-ray systems. Images have been interpolated to the identical pixel size of 200 by 200 µm. Regions of Interest (ROIs) representing the patient image (Fig. 9(a)), and ROIs representing lung tissue, bone tissue in the ribs and in the spine, and soft tissue represented by the liver region were defined using semi-automatic segmentation (Fig. 9(b)) on the de-noised patient image. ROIs were then transferred to all other images after registration using normalized mutual information with 5 degrees of freedom (allowing for translation in x and y, z-rotation, scaling in x and y). Thus, ROIs defined identically described the tissues on all registered images allowing direct comparison of pixel values.

To demonstrate differences in image processing algorithms resulting in different image characteristics the phantom has been imaged on three additional clinical systems using portable indirect CsI based flat panel detectors. Application specialists of the respective vendors were asked to implement image processing settings optimized or recommended for neonatal or baby chest imaging. Since standard protocols were different and used 54 to 70 kVp, and 12.5 to 27 µGy incident air kerma (IAK), 66 kV was selected as a common basis. mAs were set on each system to result in 26 to 27 µGy IAK which corresponded to 0.63 mAs on System 2, and 1.0 mAs on all others, respectively. Since systems 3 and 4 store images with small pixel values corresponding to low attenuation, as opposed to the other systems where small pixel values correspond to low signal, these images were inverted. To allow direct comparison of the phantom images of the four different systems (Agfa DX-G/S as the reference system, and system 2 to 4) using different image latitudes and levels in the pixel values, all images were normalized to a median pixel value and a latitude of 1024 respectively. For this purpose, the image latitude was defined as 2 times the inter-percentile value between 10^th^ and 90^th^ percentile (p10 and p90), i.e. L = 2(p90-p10).

All image processing (interpolation, registration, segmentation, normalization, and generation of histograms) was performed using AnalyzeAVW^22,23^ (Biomedical Image Resource, Rochester, USA). Calculation of percentiles was performed in Microsoft Excel 16.16 (Microsoft, Redmond, USA) using cumulated histograms from AnalyzeAVW applying linear interpolation.

### Ethics declaration and compliance

Methods applied in this work were carried out in accordance of guidelines and regulations. A positive vote (1514/2019) from the Ethics Committee of the Medical University of Vienna has been obtained for retrospective use of anonymized patient images as printing template, experimental protocols, and use of the phantom in system comparison/evaluation and optimization of pediatric radiographic procedures. The need for renewed informed consent forms was waived by the Ethics Committee since no interaction/interference with patients or treatment occurred, image data was anonymized and only selection criterion was that the image represented a typical neonatal case.

## Data Availability

The datasets generated during and analysed in the current study are available from the corresponding author on reasonable request. The STL file of the phantom is available upon request for academic collaborations.
